# Supplementary material for: Application of Ligilactobacillus salivarius CECT5713 to Achieve Term Pregnancies in Women with Repetitive Abortion or Infertility of Unknown Origin by Microbiological and Immunological Modulation of the Vaginal Ecosystem
Source: Nutrients. 2021 Jan 6;13(1):162. doi: 10.3390/nu13010162 (PMC7825435; doi:10.3390/nu13010162)
Supplement: Supplementary file 1 [file nutrients-13-00162-s001.zip › Supplementary Figure S2 (1).pptx]

## Slide 1
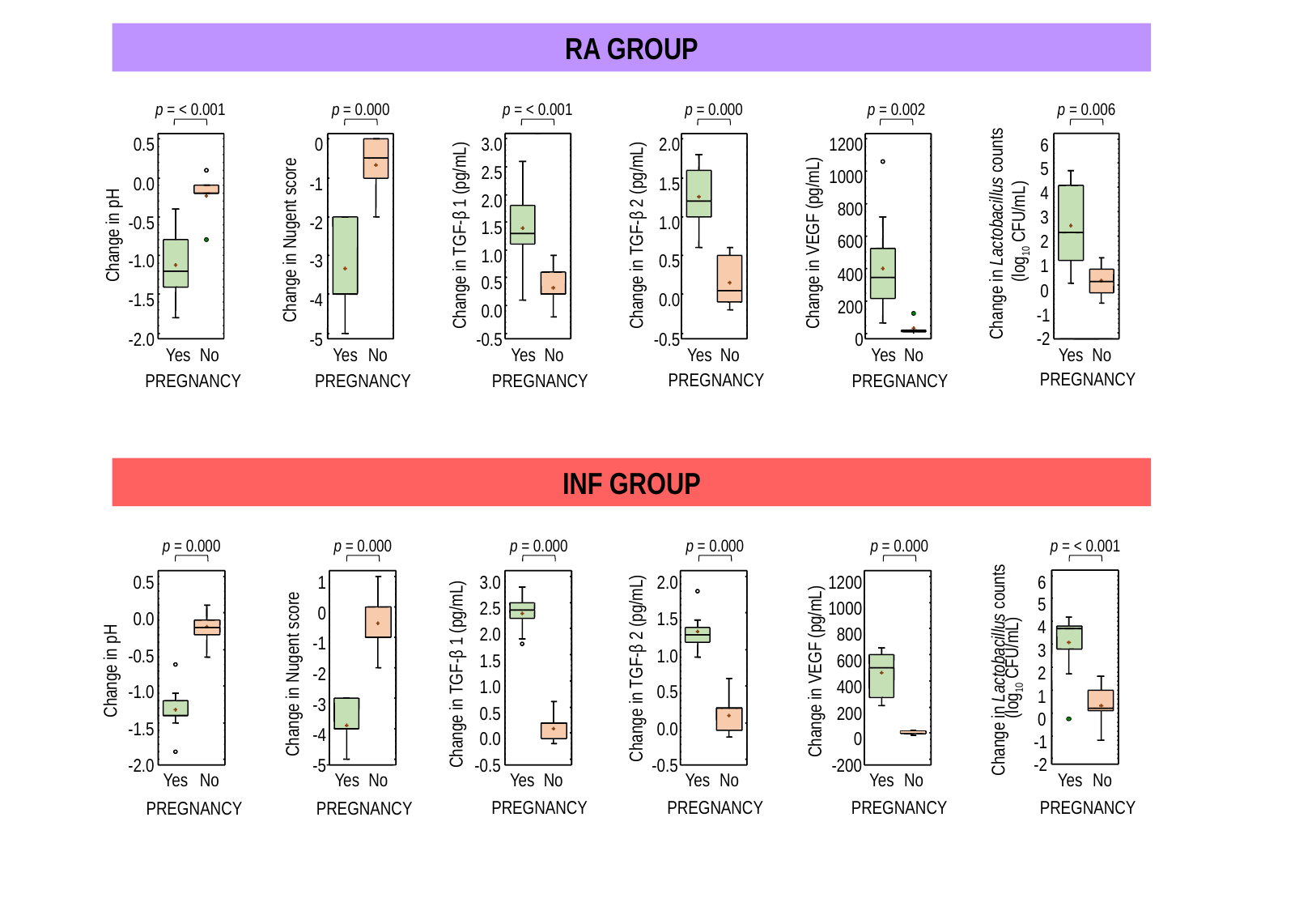

RA GROUP
p = < 0.001
p = 0.000
p = < 0.001
p = 0.000
p = 0.002
p = 0.006
6
5
4
3
Change in Lactobacillus counts
 (log10 CFU/mL)
2
1
0
-1
-2
Yes
No
PREGNANCY
3.0
2.5
2.0
1.5
Change in TGF-β 1 (pg/mL)
1.0
0.5
0.0
-0.5
Yes
No
PREGNANCY
0.5
0.0
-0.5
Change in pH
-1.0
-1.5
-2.0
Yes
No
PREGNANCY
0
-1
-2
Change in Nugent score
-3
-4
-5
Yes
No
PREGNANCY
2.0
1.5
1.0
Change in TGF-β 2 (pg/mL)
0.5
0.0
-0.5
Yes
No
PREGNANCY
1200
1000
800
600
Change in VEGF (pg/mL)
400
200
0
Yes
No
PREGNANCY
INF GROUP
p = 0.000
p = 0.000
p = 0.000
p = 0.000
p = 0.000
p = < 0.001
6
5
4
3
Change in Lactobacillus counts
(log10 CFU/mL)
2
1
0
-1
-2
Yes
No
PREGNANCY
0.5
0.0
-0.5
Change in pH
-1.0
-1.5
-2.0
Yes
No
PREGNANCY
1
0
-1
-2
Change in Nugent score
-3
-4
-5
Yes
No
PREGNANCY
3.0
2.5
2.0
1.5
Change in TGF-β 1 (pg/mL)
1.0
0.5
0.0
-0.5
Yes
No
PREGNANCY
2.0
1.5
1.0
Change in TGF-β 2 (pg/mL)
0.5
0.0
-0.5
Yes
No
PREGNANCY
1200
600
400
200
0
-200
Yes
No
1000
800
Change in VEGF (pg/mL)
PREGNANCY
