# Supplementary material for: Application of Ligilactobacillus salivarius CECT5713 to Achieve Term Pregnancies in Women with Repetitive Abortion or Infertility of Unknown Origin by Microbiological and Immunological Modulation of the Vaginal Ecosystem
Source: Nutrients. 2021 Jan 6;13(1):162. doi: 10.3390/nu13010162 (PMC7825435; doi:10.3390/nu13010162)
Supplement: Supplementary file 1 [file nutrients-13-00162-s001.zip › Supplementary Figure S3 (1).pptx]

## Slide 1
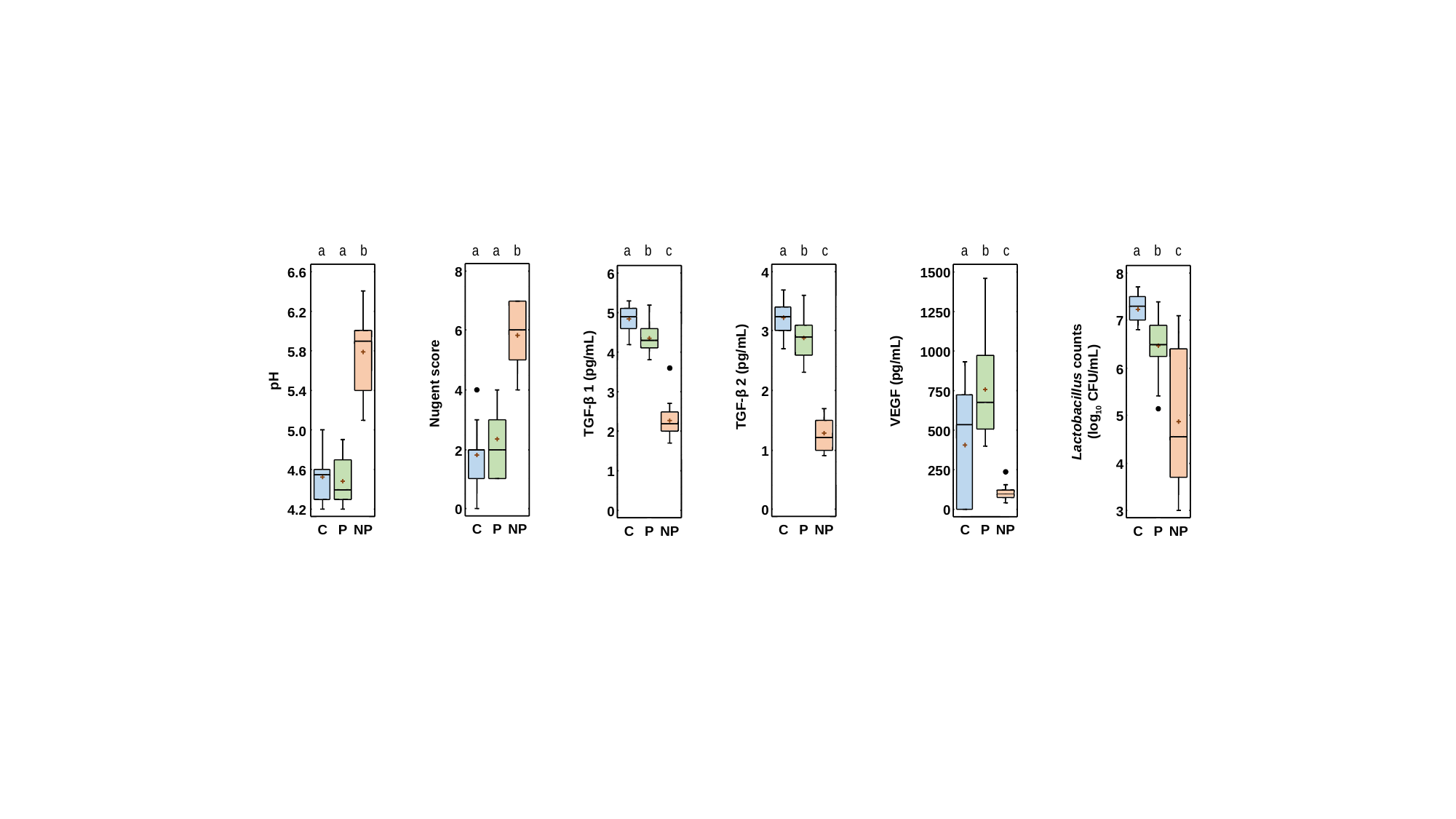

a
a
b
a
a
b
a
b
c
a
b
c
a
b
c
a
b
c
8
6
Nugent score
4
2
0
C
P
NP
6.6
6.2
5.8
pH
5.4
5.0
4.6
4.2
C
P
NP
2
1
0
C
P
NP
4
3
TGF-β 2 (pg/mL)
1500
1250
1000
VEGF (pg/mL)
750
500
250
0
C
P
NP
6
5
4
TGF-β 1 (pg/mL)
3
2
1
0
C
P
NP
8
7
6
Lactobacillus counts
(log10 CFU/mL)
5
4
3
C
P
NP
