# Supplementary material for: Application of Ligilactobacillus salivarius CECT5713 to Achieve Term Pregnancies in Women with Repetitive Abortion or Infertility of Unknown Origin by Microbiological and Immunological Modulation of the Vaginal Ecosystem
Source: Nutrients. 2021 Jan 6;13(1):162. doi: 10.3390/nu13010162 (PMC7825435; doi:10.3390/nu13010162)
Supplement: Supplementary file 1 [file nutrients-13-00162-s001.zip › Supplementary Table 1 (1).docx]

**Supplementary Table 1.** Relative frequencies, medians and interquartile ranges (IQR) of the most abundant bacterial phyla (grey shadow) and genera detected in CVL samples from women who were able to complete a full-term pregnancy (*n* = 15) and of those who did not (n = 6) among the women that had a history of repetitive abortion (RA group; *n* = 21.

|  | **Probiotic intervention resulted in pregnancy** | | | |  |
| --- | --- | --- | --- | --- | --- |
|  | **Yes (*n* = 15)** | | **No (*n* = 6)** | |  |
| **Phylum (bold)/**Genus | **n (%)^1^** | **Median (IQR)** | **n (%)** | **Median (IQR)** | ***p*-value**^2^ |
| ***Firmicutes*** | 15 (100) | 97.71 (71.86 ‒ 99.40) | 6 (100) | 96.79 (78.33 ‒ 97.47) | 0.680 |
| *Lactobacillus* | 15 (100) | 90.54 (57.22 ‒ 97.99) | 6 (100) | 94.85 (76.35 ‒ 97.15) | 0.970 |
| *Staphylococcus* | 14 (93) | 0.45 (0.04 ‒ 1.74) | 5 (83) | 0.35 (0.04 ‒ 0.66) | 0.700 |
| *Streptococcus* | 11 (73) | 0.02 (< 0.01 ‒ 0.33) | 3 (50) | < 0.01 (< 0.01 ‒ 0.56) | 0.610 |
| *Finegoldia* | 13 (87) | 0.25 (0.05 ‒ 1.03) | 5 (83) | 0.14 (0.09 ‒ 0.20) | 0.410 |
| *Peptoniphilus* | 11 (73) | 0.15 (0.01 ‒ 0.83) | 5 (83) | 0.05 (0.02 ‒ 0.10) | 0.510 |
| *Anaerococcus* | 13 (87) | 0.18 (0.06 ‒ 0.45) | 5 (83) | 0.07 (0.05 ‒ 0.09) | 0.150 |
| ***Actinobacteria*** | 15 (100) | 0.39 (0.06 ‒ 6.26) | 6 (100) | 0.22 (0.09 ‒ 20.50) | 1.000 |
| *Gardnerella* | 8 (53) | 0.01 (< 0.01 ‒ 0.16) | 3 (50) | 0.03 (< 0.01 ‒ 0.10) | 0.930 |
| *Bifidobacterium* | 8 (53) | 0.01 (< 0.01 ‒ 0.08) | 1 (17) | < 0.01 (< 0.01 ‒ < 0.01) | 0.300 |
| *Atopobium* | 6 (40) | < 0.01 (< 0.01 ‒ 0.07) | 1 (17) | < 0.01 (< 0.01 ‒ < 0.01) | 0.270 |
| ***Proteobacteria*** | 15 (100) | 0.28 (0.09 ‒ 4.71) | 6 (100) | 0.31 (0.15 ‒ 0.58) | 0.910 |
| *Escherichia/Shigella* | 7 (47) | < 0.01 (< 0.01 ‒ 0.05) | 2 (33) | < 0.01 (< 0.01 ‒ 0.01) | 0.410 |
| ***Bacteroidetes*** | 13 (87) | 0.3 (0.07 ‒ 1.44) | 5 (83) | 0.09 (0.04 ‒ 0.16) | 0.330 |
| *Prevotella* | 10 (67) | 0.07 (< 0.01 ‒ 0.58) | 5 (83) | 0.05 (0.03 ‒ 0.14) | 0.750 |
| ***Tenericutes*** | 3 (20) | < 0.01 (< 0.01 ‒ < 0.01) | 2 (33) | < 0.01 (< 0.01 ‒ < 0.01) | 0.640 |
| **Minor phyla** | 15 (100) | 0.21 (0.06 ‒ 0.66) | 6 (100) | 0.14 (0.12 ‒ 0.22) | 0.730 |
| Minor genera | 15 (100) | 0.89 (0.22 ‒ 5.39) | 6 (100) | 0.74 (0.24 ‒ 2.76) | 0.680 |
| Unclassified_genera | 15 (100) | 0.08 (0.06 ‒ 0.77) | 6 (100) | 0.20 (0.09 ‒ 0.29) | 0.730 |

^1^ n (%): number of samples in which the phylum/genus was detected (relative frequency of detection).

^2^ Kruskal-Wallis rank tests with Bonferroni correction.
