# Supplementary material for: Application of Ligilactobacillus salivarius CECT5713 to Achieve Term Pregnancies in Women with Repetitive Abortion or Infertility of Unknown Origin by Microbiological and Immunological Modulation of the Vaginal Ecosystem
Source: Nutrients. 2021 Jan 6;13(1):162. doi: 10.3390/nu13010162 (PMC7825435; doi:10.3390/nu13010162)
Supplement: Supplementary file 1 [file nutrients-13-00162-s001.zip › Supplementary Table 2 (1).docx]

**Supplementary Table 2.** Relative frequencies, medians and interquartile ranges (IQR) of the most abundant bacterial phyla (grey shadow) and genera detected in CVL samples from women who were able to complete a full-term pregnancy (*n* = 10) and of those who did not (*n* = 13) among the women with infertility of unknown origin (INF group; *n* = 23).

|  | **Probiotic intervention resulted in pregnancy** | | | | | | |  |
| --- | --- | --- | --- | --- | --- | --- | --- | --- |
|  | **Yes (*n* = 10)** | | **No (*n* = 13)** | | |  | | |
| **Phylum/**Genus | **n (%)^1^** | **Median (IQR)** | | **n (%)** | **Median (IQR)** | | ***p*-value**^2^ | |
| ***Firmicutes*** | 10 (100%) | 86.26 (43.17 ‒ 98.43) | | 13 (100%) | 94.98 (54.24 ‒ 98.91) | | 0.690 | |
| *Lactobacillus* | 10 (100%) | 72.94 (0.63 ‒ 94.16) | | 13 (100%) | 71.95 (43.61 ‒ 92.66) | | 0.980 | |
| *Streptococcus* | 6 (60%) | 0.03 (< 0.01 ‒ 0.26) | | 10 (77%) | 0.62 (0.02 ‒ 2.28) | | 0.360 | |
| *Staphylococcus* | 9 (90%) | 0.78 (0.12 ‒ 4.72) | | 13 (100%) | 0.43 (0.28 ‒ 5.62) | | 0.740 | |
| *Finegoldia* | 6 (60%) | 0.04 (< 0.01 ‒ 0.67) | | 11 (85%) | 0.35 (0.12 ‒ 3.05) | | 0.160 | |
| *Enterococcus* | 4 (40%) | < 0.01 (< 0.01 ‒ 0.03) | | 8 (62%) | 0.03 (< 0.01 ‒ 0.22) | | 0.340 | |
| *Corynebacterium* | 9 (90%) | 0.03 (0.02 ‒ 0.47) | | 10 (77%) | 0.03 (0.02 ‒ 0.57) | | 0.900 | |
| *Peptoniphilus* | 6 (60%) | 0.05 (< 0.01 ‒ 0.92) | | 11 (85%) | 0.16 (0.03 ‒ 3.31) | | 0.360 | |
| *Anaerococcus* | 8 (80%) | 0.07 (0.01 ‒ 1.07) | | 10 (77%) | 0.20 (0.03 ‒ 1.77) | | 0.550 | |
| *Dialister* | 7 (70%) | 0.04 (0.01 ‒ 0.27) | | 11 (85%) | 0.22 (0.02 ‒ 1.15) | | 0.370 | |
| *Erysipelotrichaceae* | 1 (10%) | < 0.01 (< 0.01 ‒ < 0.01) | | 2 (15%) | < 0.01 (< 0.01 ‒ < 0.01) | | 0.670 | |
| *Veillonella* | 4 (40%) | < 0.01 (< 0.01 ‒ 0.02) | | 5 (38%) | < 0.01 (< 0.01 ‒ 0.05) | | 0.970 | |
| ***Actinobacteria*** | 10 (100%) | 6.76 (0.07 ‒ 41.08) | | 13 (100%) | 1.28 (0.17 ‒ 9.18) | | 0.740 | |
| *Gardnerella* | 4 (40%) | < 0.01 (< 0.01 ‒ 11.81) | | 5 (38%) | < 0.01 (< 0.01 ‒ 0.01) | | 0.700 | |
| *Bifidobacterium* | 4 (40%) | < 0.01 (< 0.01 ‒ 0.03) | | 5 (38%) | < 0.01 (< 0.01 ‒ 0.07) | | 0.800 | |
| *Atopobium* | 6 (60%) | 0.03 (< 0.01 ‒ 0.60) | | 7 (54%) | 0.01 (< 0.01 ‒ 0.08) | | 0.670 | |
| ***Bacteroidetes*** | 10 (100%) | 0.69 (0.13 ‒ 1.32) | | 12 (92%) | 1.68 (0.04 ‒ 4.63) | | 0.830 | |
| *Prevotella* | 8 (80%) | 0.42 (0.04 ‒ 1.12) | | 11 (88%) | 1.19 (0.01 ‒ 4.63) | | 0.690 | |
| ***Proteobacteria*** | 9 (90%) | 0.14 (0.09 ‒ 0.40) | | 13 (100%) | 0.32 (0.11 ‒ 0.83) | | 0.310 | |
| *Haemophilus* | 3 (30%) | < 0.01 (< 0.01 ‒ 0.01) | | 6 (46%) | < 0.01 (< 0.01 ‒ 0.05) | | 0.440 | |
| ***Tenericutes*** | 5 (50%) | 0.14 (< 0.01 ‒ 2.05) | | 5 (38%) | < 0.01 (< 0.01 ‒ 0.20) | | 0.520 | |
| *Ureaplasma* | 5 (50%) | 0.14 (< 0.01 ‒ 2.05) | | 5 (38.46%) | < 0.01 (< 0.01 ‒ 0.20) | | 0.520 | |
| Minor_phyla | 10 (100%) | 0.11 (0.08 ‒ 0.64) | | 13 (100%) | 0.51 (0.10 ‒ 1.33) | | 0.420 | |
| *Sneathia* | 2 (20%) | < 0.01 (< 0.01 ‒ < 0.01) | | 0 (0%) | - | | - | |
| Minor_genera | 10 (100%) | 1.8 (0.28 ‒ 13.15) | | 13 (100%) | 2.21 (0.45 ‒ 6.44) | | 0.930 | |
| Unclassified_genera | 10 (100%) | 0.13 (0.04 ‒ 0.24) | | 13 (100%) | 0.15 (0.08 ‒ 0.4) | | 0.690 | |

^1^ n (%): number of samples in which the phylum/genus was detected (relative frequency of detection).

^2^ Kruskal-Wallis rank tests with Bonferroni correction.
