# Supplementary material for: Application of Ligilactobacillus salivarius CECT5713 to Achieve Term Pregnancies in Women with Repetitive Abortion or Infertility of Unknown Origin by Microbiological and Immunological Modulation of the Vaginal Ecosystem
Source: Nutrients. 2021 Jan 6;13(1):162. doi: 10.3390/nu13010162 (PMC7825435; doi:10.3390/nu13010162)
Supplement: Supplementary file 1 [file nutrients-13-00162-s001.zip › Supplementary Table 3 (1).docx]

**Supplementary Table 3.** Differences in the baseline characteristics and effect of the probiotic intervention with *L. salivarius* CECT5713 on the vaginal parameters of all women who were able to complete a full-term pregnancy (*n* = 25) and of those who did not (*n* = 19) among all participants from both RA and INF groups (*n* = 44).

|  | **Probiotic intervention resulted in pregnancy** | |  |
| --- | --- | --- | --- |
|  | **Yes (*n* = 25)** | **No (*n* = 19)** |  |
| **Parameter** | **[mean (95% CI)]** | **[mean (95% CI)]** | ***p-*value^1^** |
| **pH** |  |  |  |
| Baseline | 5.69 (5.57 ‒ 5.81) | 5.99 (5.85 ‒ 6.13) | 0.024 |
| Post-intervention | 4.48 (4.40 ‒ 4.56) | 5.78 (5.69 ‒ 5.87) | 0.000 |
| Change | -1.20 (-1.29 ‒ -1.12) | -0.21 (-0.31 ‒ -0.10) | 0.000 |
| *p*-value^3^ | 0.000 | < 0.001 |  |
| **Nugent score** |  |  |  |
| Baseline | 5.92 (5.51 ‒ 6.33) | 6.42 (5.95 ‒ 6.89) | 0.258 |
| Post-intervention | 2.36 (2.06 ‒ 2.66) | 5.84 (5.50 ‒ 6.19) | 0.000 |
| Change | -3.56 (-3.82 ‒ -3.30) | -0.58 (-0.88 ‒ -0.28) | 0.000 |
| *p*-value^3^ | 0.000 | 0.004 |  |
| **TGF-β 1 (pg/mL)** |  |  |  |
| Baseline | 2.60 (1.50 – 3.70) | 4.06 (2.81 ‒ 5.32) | 0.217 |
| Post-intervention | 4.36 (4.24 ‒ 4.47) | 2.27 (2.14 ‒ 2.41) | 0.000 |
| Change | 1.76 (1.60 ‒ 1.91) | -1.79 (-0.03 ‒ 0.33) | 0.000 |
| *p*-value^3^ | 0.000 | 0.193 |  |
| **TGF-β 2 (pg/mL)** |  |  |  |
| Baseline | 1.63 (1.56 ‒ 1.70) | 1.16 (1.08 ‒ 1.24) | 0.000 |
| Post-intervention | 2.88 (2.80 ‒ 2.96) | 1.27 (1.18 ‒ 1.37) | 0.000 |
| Change | 1.25 (1.17 ‒ 1.33) | 0.12 (0.02 ‒ 0.21) | 0.000 |
| *p*-value^3^ | 0.000 | 0.092 |  |
| **VEGF (pg/mL)** |  |  |  |
| Baseline | 329 (304 ‒ 354) | 90 (61 ‒ 119) | 0.000 |
| Post-intervention | 755 (693 ‒ 817) | 103 (32 ‒ 174) | 0.000 |
| Change | 426 (378 ‒ 473) | 13 (-41 ‒ 68) | 0.000 |
| *p*-value^3^ | 0.000 | 0.068 |  |
| **Lactobacilli presence [n (%)]** |  |  |  |
| Baseline | 12 (48) | 6 (32) | 0.359^2^ |
| Post-intervention | 25 (100) | 10 (53) | < 0.001^2^ |
| Change | 13 (52) | 4 (21) | 0.060^2^ |
| **Lactobacilli counts (log_10_ CFU/mL)** | |  |  |
| Initial | 4.99 (4.48 ‒ 5.50) | 5.88 (5.16 ‒ 6.60) | 0.150 |
| Final | 6.47 (6.21 ‒ 6.73) | 4.87 (4.45 ‒ 5.28) | 0.000 |
| Change | 2.67 (2.26 ‒ 3.08) | 0.26 (-0.39 ‒ 0.90) | < 0.001 |
| *p*-value^3^ | 0.000 | 0.360 |  |
| ***L. salivarius* qPCR** **[n (%)]** |  |  |  |
| Initial | nd | nd | - |
| Final | 25 (100) | 7 (37) | < 0.001^2^ |
| ***L. salivarius* qPCR (log_10_ copies/mL)^4^** | |  |  |
| Initial | - | - | - |
| Final | 6.70 (6.52 ‒ 6.89) | 3.16 (2.81 ‒ 3.51) | 0.000 |

^1^ One-way ANOVA tests were used to evaluate differences in mean values between groups, except for lactobacilli presence.

^2^ Fisher exact probability test for a 2×2 contingency table.

^3^ One-way repeated measures ANOVA tests were used to determine whether there was a change in each group of participants when comparing the baseline and post-intervention parameters.

^4^ Mean (95% CI) of *L. salivarius* qPCR (copies/mL) i n positive samples.
