# Supplementary material for: Application of Ligilactobacillus salivarius CECT5713 to Achieve Term Pregnancies in Women with Repetitive Abortion or Infertility of Unknown Origin by Microbiological and Immunological Modulation of the Vaginal Ecosystem
Source: Nutrients. 2021 Jan 6;13(1):162. doi: 10.3390/nu13010162 (PMC7825435; doi:10.3390/nu13010162)
Supplement: Supplementary file 1 [file nutrients-13-00162-s001.zip › Supplementary Table 4 (1).docx]

**Supplementary Table 4.** Relative frequencies, medians and interquartile ranges (IQR) of the most abundant bacterial phyla (grey shadow) and genera detected in CVL samples from women who were able to complete a full-term pregnancy (*n* = 25) and of those who did not (*n* = 19) among women with a history of reproductive failure, because either of recurrent miscarriage (RA group) or infertility of unknown origin (INF groups) (*n* = 44).

|  | **Probiotic intervention resulted in pregnancy** | | | | |  |
| --- | --- | --- | --- | --- | --- | --- |
|  | **Yes (*n* = 25)** | | **No (*n* = 19)** | |  | |
| **Phylum/**Genus | **n (%)^1^** | **Median (IQR)** | **n (%)** | **Median (IQR)** | ***p*-value^2^** | |
| ***Firmicutes*** | 25 (100) | 89.96 (70.34 ‒ 99.35) | 19 (100) | 95.18 (55.01 ‒ 98.22) | 0.830 | |
| *Lactobacillus* | 25 (100) | 83.34 (28.22 ‒ 97.49) | 19 (100) | 90.21 (45.25 ‒ 95.41) | 0.640 | |
| *Staphylococcus* | 23 (92) | 0.75 (0.06 ‒ 2.08) | 18 (95) | 0.43 (0.08 ‒ 4.34) | 0.700 | |
| *Streptococcus* | 17 (68) | 0.02 (< 0.01 ‒ 0.32) | 13 (68) | 0.47 (< 0.01 ‒ 2.04) | 0.300 | |
| *Finegoldia* | 19 (76) | 0.13 (0.03 ‒ 0.93) | 16 (84) | 0.21 (0.10 ‒ 0.86) | 0.490 | |
| *Peptoniphilus* | 17 (68) | 0.10 (< 0.01 ‒ 1.03) | 16 (84) | 0.09 (0.02 ‒ 0.61) | 0.720 | |
| *Enterococcus* | 7 (28) | < 0.01 (< 0.01 ‒ 0.02) | 11 (58) | 0.03 (< 0.01 ‒ 0.23) | 0.052 | |
| *Anaerococcus* | 21 (84) | 0.14 (0.02 ‒ 0.57) | 15 (79) | 0.08 (0.04 ‒ 1.29) | 0.890 | |
| ***Actinobacteria*** | 25 (100) | 0.43 (0.07 ‒ 14.21) | 19 (100) | 0.64 (0.13 ‒ 18.21) | 0.850 | |
| *Gardnerella* | 12 (48) | < 0.01 (< 0.01 ‒ 0.24) | 8 (42) | < 0.01 (< 0.01 ‒ 0.05) | 0.530 | |
| *Bifidobacterium* | 12 (48) | < 0.01 (< 0.01 ‒ 0.06) | 6 (32) | < 0.01 (< 0.01 ‒ 0.05) | 0.590 | |
| *Atopobium* | 12 (48) | < 0.01 (< 0.01 ‒ 0.11) | 8 (42) | < 0.01 (< 0.01 ‒ 0.07) | 0.580 | |
| ***Proteobacteria*** | 24 (96) | 0.23 (0.09 ‒ 0.69) | 19 (100) | 0.32 (0.11 ‒ 0.74) | 0.510 | |
| *Escherichia/Shigella* | 13 (52) | < 0.01 (< 0.01 ‒ 0.03) | 4 (21) | < 0.01 (< 0.01 ‒ < 0.01) | 0.043 | |
| ***Bacteroidetes*** | 23 (92) | 0.31 (0.07 ‒ 1.34) | 17 (89) | 0.17 (0.04 ‒ 3.16) | 0.830 | |
| *Prevotella* | 18 (72) | 0.11 (< 0.01 ‒ 0.71) | 16 (84) | 0.06 (0.02 ‒ 2.28) | 0.660 | |
| *Tenericutes* | 8 (32) | < 0.01 (< 0.01 ‒ 0.28) | 7 (37) | < 0.01 (< 0.01 ‒ 0.20) | 0.910 | |
| Minor phyla | 25 (100) | 0.12 (0.07 ‒ 0.67) | 19 (100) | 0.21 (0.11 ‒ 1.29) | 0.270 | |
| Minor genera | 25 (100) | 0.91 (0.27 ‒ 5.15) | 19 (100) | 1.81 (0.40 ‒ 5.07) | 0.870 | |
| Unclassified_genera | 25 (100) | 0.11 (0.05 ‒ 0.66) | 19 (100) | 0.15 (0.08 ‒ 0.36) | 0.590 | |

^1^ n (%): number of samples in which the phylum/genus was detected (relative frequency of detection).

^2^ Kruskal-Wallis rank tests with Bonferroni correction.
