# Supplementary figures and images for: Application of Ligilactobacillus salivarius CECT5713 to Achieve Term Pregnancies in Women with Repetitive Abortion or Infertility of Unknown Origin by Microbiological and Immunological Modulation of the Vaginal Ecosystem
Source: Nutrients. 2021 Jan 6;13(1):162. doi: 10.3390/nu13010162 (PMC7825435; doi:10.3390/nu13010162)

## Slide 1
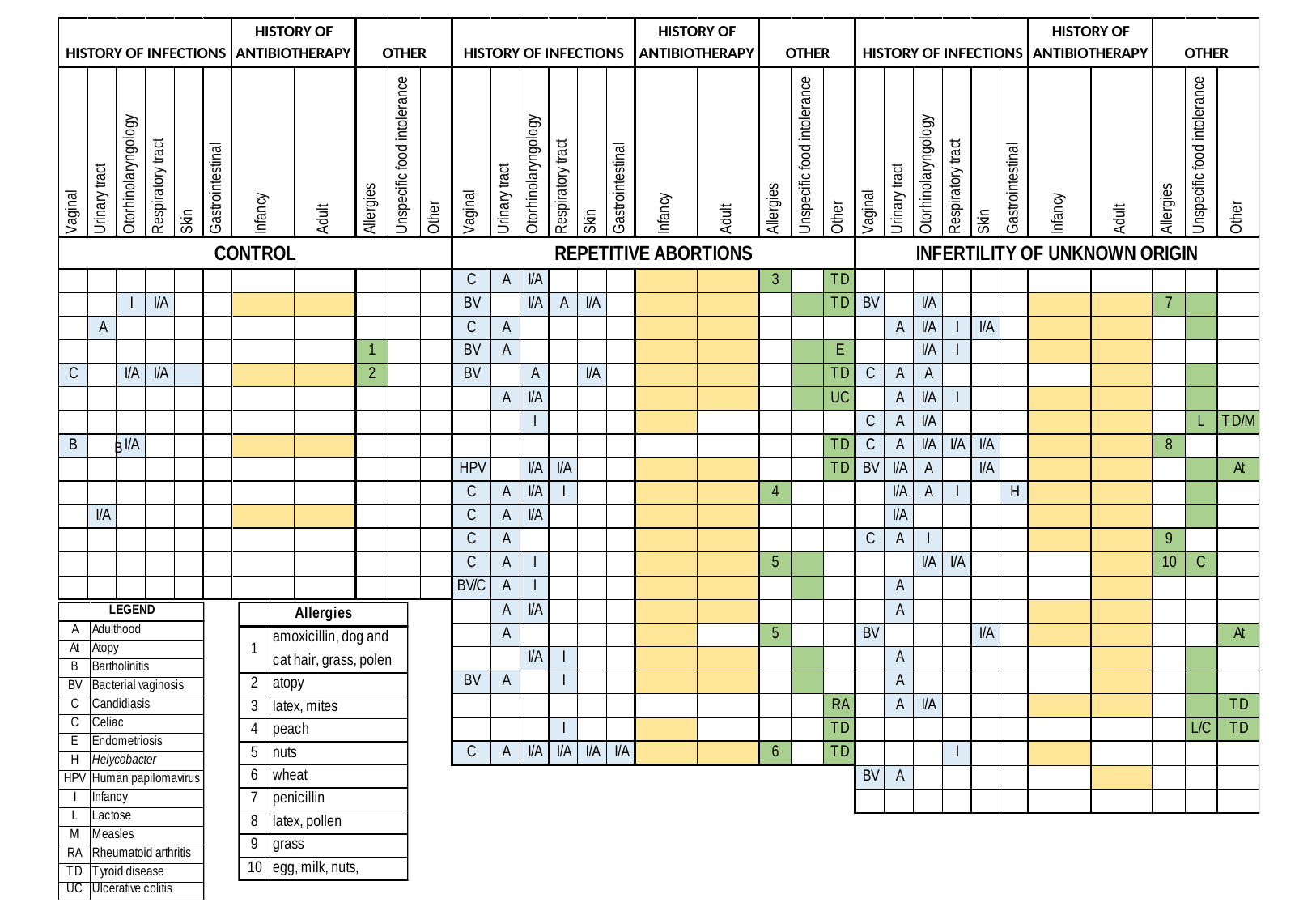

B

Supplement: Supplementary file 1 [file nutrients-13-00162-s001.zip › Supplementary Figure S1 (2).pptx]
